# Automatic Recognition of Human Interaction via Hybrid Descriptors and Maximum Entropy Markov Model Using Depth Sensors

**DOI:** 10.3390/e22080817

**Published:** 2020-07-26

**Authors:** Ahmad Jalal, Nida Khalid, Kibum Kim

**Affiliations:** 1 Department of Computer Science, Air University, Islamabad 44000, Pakistan; ahmadjalal@mail.au.edu.pk (A.J.); 190115@students.au.edu.pk (N.K.); 2 Department of Human-Computer Interaction, Hanyang University, Ansan 15588, Korea

**Keywords:** cross entropy, depth sensors, Gaussian mixture model, maximum entropy Markov model

## Abstract

Automatic identification of human interaction is a challenging task especially in dynamic environments with cluttered backgrounds from video sequences. Advancements in computer vision sensor technologies provide powerful effects in human interaction recognition (HIR) during routine daily life. In this paper, we propose a novel features extraction method which incorporates robust entropy optimization and an efficient Maximum Entropy Markov Model (MEMM) for HIR via multiple vision sensors. The main objectives of proposed methodology are: (1) to propose a hybrid of four novel features—i.e., spatio-temporal features, energy-based features, shape based angular and geometric features—and a motion-orthogonal histogram of oriented gradient (MO-HOG); (2) to encode hybrid feature descriptors using a codebook, a Gaussian mixture model (GMM) and fisher encoding; (3) to optimize the encoded feature using a cross entropy optimization function; (4) to apply a MEMM classification algorithm to examine empirical expectations and highest entropy, which measure pattern variances to achieve outperformed HIR accuracy results. Our system is tested over three well-known datasets: SBU Kinect interaction; UoL 3D social activity; UT-interaction datasets. Through wide experimentations, the proposed features extraction algorithm, along with cross entropy optimization, has achieved the average accuracy rate of 91.25% with SBU, 90.4% with UoL and 87.4% with UT-Interaction datasets. The proposed HIR system will be applicable to a wide variety of man–machine interfaces, such as public-place surveillance, future medical applications, virtual reality, fitness exercises and 3D interactive gaming.

## 1. Introduction

Human interaction recognition (HIR) deals with the understanding of communication taking place between a human and an object or other persons [1]. HIR includes an understanding of various actions, such as social interaction, person to person talking, meeting or greeting in the form of a handshake or a hug and the performance of inappropriate actions, such as fighting, kicking or punching each other. There are many different kinds of interactions that can easily be identified by human observations. However, in many situations, personal human observation of some actions is impractical due to the cost of resources and also to hazardous environments. For example, in the case of smart rehabilitation, it is more suitable for a machine to monitor a patient’s daily routine rather than for a human to constantly observe a patient (24/7) [2]. Similarly, in the case of video surveillance, it is more appropriate to monitor human actions via sensor devices, especially in places where risk factors and suspicious activities are involved.

Due to a wide variety of applications, HIR has gained much attention in recent years. These applications include public security surveillance, e-health care, smart homes, assistive robots and sports assistance [3] each of which requires an efficient understanding and identification of discrete human movements [4,5,6,7,8,9]. Many HIR systems have been proposed to tackle problems faced in activity monitoring in healthcare, rehabilitation, surveillance and many other situations. Reliable and accurate monitoring is essential in order to monitor the progress of patients in physical therapy and rehabilitation centers, to detect potential and actual dangers, such as falls and thefts, and to prevent mishaps and losses due to lack of attention [10]. HIR systems are also proposed for security reasons [11], such as a Fuzzy logic based human activity recognition system, which was proposed in [12]. A Hidden Markov Model [HMM] based HIR system was proposed for surveillance [13]. A random forest based HIR system for smart home and elderly care was proposed by H. Xu et al. [14]. A neural network-based HIR system was presented by S. Chernbumroong for assisted living [15]. Clearly, HIR systems are in demand and highly applicable in many daily life domains.

Motivated by the applications of HIR systems in daily life, we proposed a robust system which is able to track human interactions and which is easy to deploy in real world applications [16]. Challenges, such as complex and cluttered background, intra-class variations and interclass similarity make it difficult to accurately recognize and distinguish between human interactions. Therefore, we aim to increase the recognition rate of human–human interactions and tackle challenges faced by recent HIR systems by incorporating depth sensors. The recognition rate of human interactions is being boosted with a recent low-cost depth sensors technology [17,18]. Depth imaging technology is getting more attention in recent years because it is providing promising results without the attachment of marker sensors [2,19,20]. HIR systems based on depth sensors are easy to deploy in daily life applications compared to systems based on wearable or marker sensors [21]. This is because wearable sensors need to be attached to the body of an individual in order to give better performance, but this creates usability and mobility problems for the wearer. The main purpose of this research work is to propose a multi-vision sensor based HIR system which consists of a hybrid of four unique features in order to achieve a better performance rate. Our system aims at giving computers sensitivity to automatically monitor, recognize and distinguish between human actions happening in respective surroundings.

Basically, HIR can be categorized into four types—i.e., human–object interaction (HOI), human–robot interaction (HRI), human–human interaction (HHI) and human–group of humans interaction (HGI). In the case of HOI, humans act, communicate and interact with various objects to perform different daily actions [22,23] such as picking-up a glass for drinking, holding a ball for throwing and taking food for eating. During HRI, a robot may be able to perform different postures, such as hand shaking, serving food and waving hands, etc. Robots in HRI can precisely predict a human’s future actions and analyze the gestures of the persons that interact with them [24]. Similarly, in HHI and HGI, a system can estimate the trajectory information of the human–human or capture the movement patterns of groups of people [25] in crowded or public areas. However, our research work is focused on human to human interaction.

In this paper, we propose a novel hybrid HIR system and entropy Markov model that examines the daily interactions of humans. The proposed model measures the spatiotemporal properties of the body’s posture and estimates an empirical expectation of pattern changes using depth sensors. For the vision (RGB or depth) filtered data, we have adopted mean filter, pixel connectivity analysis and Otsu’s thresholding method. For hybrid descriptor features, we proposed four types of features characteristics as follows:

- Space and time based—i.e., spatio-temporal features—in which displacement measurements between key human body points are recognized as temporal features. Intensity changes along the curved body points of silhouettes are taken as spatial features.
- Motion-orthogonal histograms of oriented gradient (MO-HOG) features are based on three different views of human silhouette. These views are projected in the form of orthogonal shape and then HOG is applied.
- Shape based angular and geometric features include angular measurements over two types of shapes—i.e., inter-silhouettes and intra-silhouette.
- Energy based features examine distinct body parts energy distribution within a silhouette.

These hybrid descriptors are fed into a Gaussian mixture model (GMM) and into fisher encoding for codebook generation and for proper discrimination among various activity classes. Then, we applied cross entropy algorithm which resulted in the optimized distribution of matrixes. Finally, the maximum entropy Markov model (MEMM) is embodied in the proposed HIR system to estimate empirical expectation and the highest entropy of different human interactions to achieve significant accuracy. Four experiments were performed using a leave-one-out cross validation method on three well-known datasets. Our proposed method acquired significant performance compared to well-known statistical state-of-the-art methods. The major contributions of this paper can be highlighted as follows:

1. We proposed to apply hybrid descriptor features of spatiotemporal characteristics, invariant properties, view-orientation as well as displacement and intra/inter angular values to distinct human interactions.
2. We introduced a combination of GMM with fisher encoding for codebook generation and optimal discrimination of features.
3. We designed cross entropy optimization and MEMM to analyze contextual information as well as to classify complex human interactions in a better way.
4. We performed experiments using three publicly available datasets and the proposed method was fully validated for the efficacy, outperforming other state-of-the-art methods, including deep learning.

The rest of the paper is organized as follows: Section 2 consists of related work in the field of HIR. Section 3 presents details of our proposed methodology. Section 4 reports the experimentation, dataset description results generation. Section 5 presents a discussion of the overall paper. Finally, Section 6 concludes the proposed research work with some future directions.

## 2. Related Work

Recently, a lot of works have been done by researchers for the development of HIR using multiple types of sensors. On the basis of methods used to capture human interactions, we categorize these sensors in our related work into three major types: (1) wearable sensor-based HIR; (2) vision sensor-based HIR and (3) Marker sensor-based HIR.

### 2.1. Wearable Sensor-Based HIR Systems

In wearable sensor-based technology, many sensors (e.g., accelerometers, gyroscopes and magnetometers) are attached to the subject’s limb and body in order to examine interactions with the surroundings [26,27,28]. In [29], A. Howedi et al. proposed a unique HIR methodology based on different entropy measures, such as Fuzzy, sample and approximate entropy. They achieved significant accuracy in entropy measurements for the detection and identification during human interactions. In [30], M. Ehatisham et al. designed an action recognition system based on K-nearest neighbors and SVM via multiple sensors, including RGB cameras, depth sensors and wearable sensors for accurate recognition of human behaviors. H. Xu et al. [31] developed a wearable sensor based HIR that extracted various feature values via Hilbert–Huang transform (HHT). HHT spectrum features include frequency, amplitude, means and energy values that are tested over PAMAP2 wearable sensor datasets. Experimental results showed that multi features approach achieved better performance for HIR.

Motivated by the application of wearable sensors in health departments, a human motion detection system based on accelerometer sensor measurements is proposed by A. Jalal et al. [32]. In order to extract features of each activity class axial components of the accelerometer are taken. After extracting features, Random forest is applied to classify interactions that result in good performance in human motion detection. In order to recognize the physical activities of humans, wearable sensors are used by M. Batool et al. in [33]. They used both the gyroscopic and accelerometer sensor data.

They extracted statistical and Mel-frequency cepstral coefficient data. A combination of particle swarm optimization (PSO) and support vector machine (SVM) resulted in a better recognition rate. In order to solve the problem of feature selection and classification of sensor data, a genetic algorithm-based approach is used by M.A. Quaid et al. [34]. Statistical and acoustic features are extracted and then features are reweighted. After reweighting the features, biological operations for crossover and mutation are applied. One self-annotated dataset is proposed in this work. Experiments on three-mark datasets proved the efficiency of proposed human behavior analysis system. Motivated by applications using wearable sensors for elderly care, S. Badar et al. proposed a wearable sensor-based activity monitoring system [35]. This system consists of inertial and motion node sensors. Three types of features, such as binary, wavelet and statistical are extracted. In order to optimize features, adaptive moment estimation (Adam) and Ada delta are applied. Experiments on two datasets are used for system evaluation. The results showed a better performance compared to other state of the art systems.

In order to recognize daily activity, a smartphone with built in accelerometer was used by A.M. Khan et al. [36]. Two types of features, such as autoregressive coefficients and signal magnitude area were extracted. Kernel discriminant analysis and Artificial Neural Network (ANN) were then used for accurately identifying the activity class. Inspired by the applications of sensors embedded in smartphones, N.A. Capela et al. [37] proposed a human activity recognition system. In this research work, sensor data were taken from patients and elderly people. Seventy-six signal features were extracted and then selected on the basis of feature selection methods. Three classifiers were used to evaluate the proposed methodology and results reveal a better rate of accuracy. Motivated by healthcare and rehabilitation-based applications for human activity recognition systems, W. Jiang et al. proposed a wearable sensor-based method [38]. They collect signals from sensors in the form of activity images. They applied deep CNN for feature extraction. Evaluation on three benchmark datasets validated the performance of their system. However, these technologies face several limitations in HIR, such as discomfort and restricted movement for subjects, due to many wires and wearable sensors that are attached to their bodies [39]. Similarly, in order to capture full-body movements, the multiple sensors that are attached to the human body, cause computational complexity in the system. Background noises picked up by wearable sensors during measurements are also incorporated in the data and these result in numerous false predictions which affect decision making [40]. Therefore, instead of relying on wearable devices, vision-based sensor technologies have started gaining global attentions as a solution in HIR studies.

### 2.2. Vision-Based HIR Systems

In vision-based HIR systems, video cameras are mounted for automated inspection of human interactions in various public areas (i.e., shopping malls, parks and roads). In [41], M. Sharif et al. proposed a human activity monitoring system. They used a fused feature algorithm technique that consists of HOG, Harlick and binary patterns. Then, a novel joint entropy-based feature selection algorithm is used along with a recognizer engine (i.e., multi-class SVM) to examine HIR behavior. In [42], O. Ouyed et al. extracted motion features from the joints of two persons involved in an interaction. They used multinomial kernel logistic regression to evaluate HIR using Set I of UT-Interaction dataset. In [43], X. Ji et al. presented a vision based HIR system using multiple stage probability fusion. They divided interaction between two persons into the start state, execution state and the end state. Through weighted fusion, better recognition accuracy rates were obtained.

S. Bibi et al. [44] proposed a multi-feature model along with median compound local binary patterns for HIR system. They monitored individual action through multi-view cameras and showed better human–human interaction recognition rates. N. Cho et al. [45] described a novel system in order to identify complex human interaction identification. Their feature descriptors contained movements at global, local and individual levels. They detected points of significance in order to identify human motion. Experiments on two publicly available datasets with a SVM classifier showed that their system produced a better accuracy rate. A human activity recognition system based on depth sensors is proposed by O.F. Ince et al. [46]. Their system, which is based on joint-angle features, can detect activities in 3D space. The Haar-wavelet transform and dimension reduction algorithm is also applied. K- nearest neighbor (KNN) is applied to recognize human actions. In order to track human interaction recognition, a wise human interaction and tracking model was proposed by M. Mahmood et al. [47]. They extracted data from spatio–temporal and angular–geometric features. They evaluated their system on three benchmark datasets and, as a result, the performance of the system was better than many state-of-the-art systems.

In order to recognize human interactions in both indoor and outdoor environments, an RGB-based HIR system was introduced by Jalal [48]. Multiple features are proposed in this research work and Convolution Neural Networks (CNN) was applied. CNN proved to be better than other state-of-the-art classifiers. N. Nguyen et al. proposed an HIR system motivated by the performance of deep learning methods [49]. Hierarchical invariant features are extracted using Independent Subspace Analysis (ISA) via three-layer CNN. Through experimentation they showed that their three-layer approach is better at recognizing human interaction in complex environments than other approaches. Motivated by the success of bag-of-words, an automated recognition system was proposed by K.N. Slimani et al. [50]. They extracted a 3D volume of spatio–temporal features. Each interaction between two persons is represented by the co-occurrence of words through their frequency. Inspired by the applications of information technology (IT) in the education sector, Jalal et al. proposed a student behavior recognition (SBR) system [51]. They extracted spatio–temporal features for identifying student–student interaction. Their tested their system against one self-annotated and one RGB dataset. In [52] depth map-based person–person interaction is recognized. Interaction is divided into body part interactions. Regression based learning is used to process each camera view then features from multiple views are combined. The efficacy of the system was evaluated on three public depth-based datasets.

These methods mentioned above are either implemented on single RGB data or have used a very small set of features. On the other hand, we propose a vision based HIR system that consists of hybrid features having generic properties for RGB as well as depth images. For experimental validation, we use two depth datasets and one RGB dataset that consist of complex interactions over indoor–outdoor environments.

### 2.3. Marker Sensor-Based HIR Systems

In marker-based HIR systems, different markers, such as light emitting diodes, infrared or reflective spheres, are attached to the human body in order to capture motion information [53]. These sensors are attached to targeted body regions, such as joints or limbs of the human body. Many researchers used marker sensors for human activity analysis, clinical diagnosis and in rehabilitation centers. For example, M.H. Khan proposed a marker sensor-based system in order to provide home based therapy for patients [54]. Markers of different colors are attached to the individual’s joints and motion information is recorded. Experiments were conducted on 10 patients which validated the performance of proposed systems. In [55], color markers are used to track foot positions. The motions of different body parts are tracked with the help of marker sensors and then interaction information between person and virtual surroundings is achieved. In order to analyze upper limb function of patients with abnormal limbs, a combination of a hand skateboard device, an IR camera and an infrared emitter is used [56]. Experiments showed that this system is easy to use and that it delivers results immediately.

In order to perform biomechanical examinations and to capture motion in sports activities, marker based optical sensors are used [57]. For system evaluation, collegiate and elite baseball pitchers are used. A Trunk Motion System (TMS) was developed by M.I. Esfahani [58]. They used Body-Worn Sensors (BWS) in their system. They measured the 3D motions of the trunk. Their system is very lightweight. They attached 12 Body-Worn Sensors on stretchable clothing. Seven actions were performed wearing BWS and the results were evaluated based on these actions. Motivated by a wide variety of applications using motion sensing in the healthcare department, a BWS based monitoring system was proposed by J. Michael et al. [59]. In order to measure the physical activities of humans, an innovative wireless system is proposed by N. Golestani et al. [60]. They proposed a magnetic induction system to track human actions. In this system, markers are attached to the joints. Successful evaluations were performed by applying laboratory measurements and deep recurrent neural network monitoring.

These sensors provide very accurate information regarding position, but they lack effectiveness in high speed motion because they cannot read and produce data on factors such as acceleration, velocity and torque. More precise results are generated via marker sensors, which provide better results in many clinical studies [61]. However, their performance was affected by surroundings such as dust, temperature changes and vibrations [62].

## 3. Proposed System Methodology

**Figure 1:**
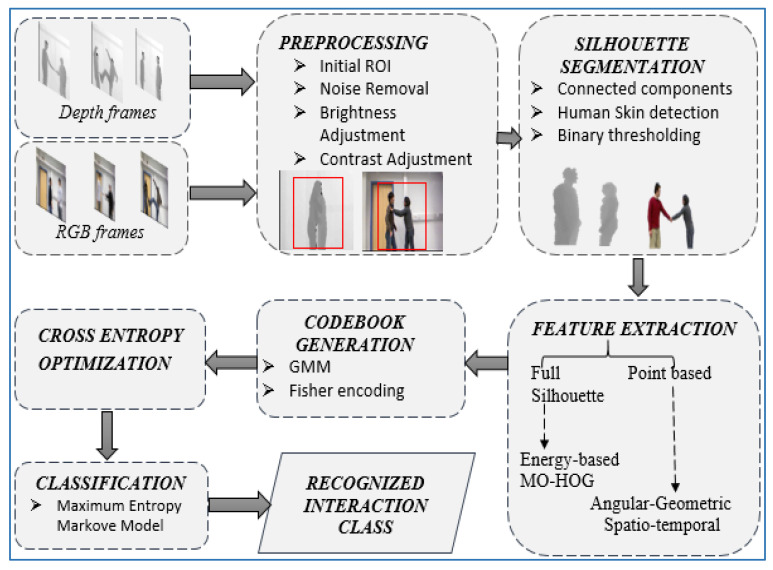
System architecture of proposed human interaction recognition model.

In this section, we describe details of each process involved in the proposed HIR system. Firstly, raw image (i.e., RGB and depth) sequences are preprocessed to remove noise. Then the segmentation algorithms are applied to extract the foregrounds from the backgrounds. Secondly, after segmentation, four different types of features are extracted as hybrid descriptor features. These feature descriptors are then fed into a codebook generation algorithm. Thirdly, cross entropy algorithms are applied to optimize the quantized codebook. Finally, experiments are performed and MEMM is used to determine each interaction class. Figure 1 shows the complete system architecture of the proposed HIR methodology.

### 3.1. Image Acquisition

During image acquisition, we start with video normalization to extract human silhouette representations by applying various techniques for noise removal, handling varying scales and contrasting distribution. For these purposes, all image sequences are first cropped to a fixed dimension to remove unnecessary areas. In order to enhance image quality, brightness and contrast, the distribution of both RGB and depth images are adjusted to make the images clearer via histogram equalization. Then, a smoothing filter is applied as mean filter [63], which calculates all mean values between a current pixel and its neighboring pixels. The mean filter of input signal *x* is given through Equation (1):

(1) $$y[i]=\frac{1}{M}.\sum_{i=0}^{M-1}x(i+j)$$

where *y* is the smoothened image, *i* and *j* are pixel values, and *M* is the window size, having a number of neighboring pixels.

### 3.2. Silhouette Representation

For robust identification of HIR, actual human interaction areas need to be extracted and to distinguish target images from clutter [64]. To extract efficient silhouette representation, we depend mainly on connected components, skin tone, region growing and color spacing [65]. Various algorithms are used for both RGB and RGB-D silhouette segmentation to improve the performance of the proposed system. We discuss this below.

#### 3.2.1. Silhouette Segmentation of RGB Images

**Figure 2:**
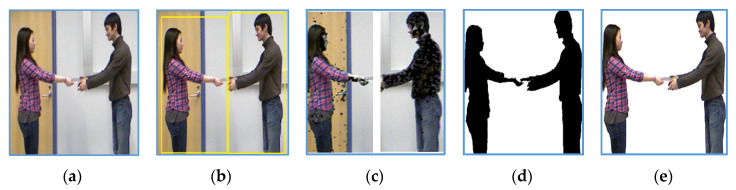
Example of RGB silhouette segmentation for an Exchanging object interaction of SBU dataset: (**a**) original image; (**b**) detected silhouettes; (**c**) skin coloring on cropped left and right silhouette; (**d**) binary thresholding over silhouettes and (**e**) segmented RGB silhouettes.

RGB silhouette segmentation is performed on the basis of pixel connectivity analysis and skin detection [66]. Initially, we detect human silhouettes where connected components in an image are found using 8-connected pixel analysis. This technique seeks to identify horizontal, vertical and diagonal connections between pixels. Human silhouettes are then defined by auto-bounding on the boxes on the basis of height and width parameters. Next, to segment silhouettes from a noisy background, we apply coloring algorithms to identify all light intensity colors, such as yellow, skin color and white. These light intensity colors are then converted from RGB to luminance, chrominance (yCbCr) color space, which is formulated as:

(2) $$\left[\begin{array}{l} Y\\ Cb\\ Cr \end{array}\right]=\left[\begin{array}{l} 16\\ 128\\ 128 \end{array}\right]+[\begin{array}{cc} 65.481 & 128.553\\ -37.797 & -74.203\\ 112 & -93.786 \end{array}\begin{array}{c} 24.966\\ 112\\ -18.214 \end{array}]\;\;\left[\begin{array}{l} R\\ G\\ B \end{array}\right]$$

where *Y* is luminance, *Cb* and *Cr* represent blue difference and red difference chrominance. After the identification of light intensity colors, they are converted to black color. Then we apply threshold-based segmentation, which works as growing regions to segment humans from the background. Full procedure of RGB silhouette identification and segmentation is shown in Figure 2.

#### 3.2.2. Silhouette Segmentation of Depth Images

For silhouette segmentation of depth images, we used Otsu’s thresholding method in which an image is divided into two classes—i.e., background class and foreground class [67]. In this method, multiple iterations with possible threshold values *T* are performed and one unique value of *T* is chosen that best separates foreground and background pixels. In order to calculate *T*, inter-class and intra-class variance are needed for analyzation. In the case of intra-class analysis, variance should be as low as possible so, it is minimized through Equation (3):

(3) $$\sigma_{w}^{2}(T)=w_{0}(T)\sigma_{0}^{2}(T)+w_{1}(T)\sigma_{1}^{2}(T)$$

where probabilities of both classes that are divided by *T*, is given by $w_{0}$ and $w_{1}$. Variances of both classes are shown by $\sigma_{0}^{2}$ and $\sigma_{1}^{2}$. On the other hand, variance between two classes—i.e., inter-class variance should be as high as possible, as shown through Equation (4):

(4) $${}_{\sigma_{b}^{2}(T)=\sigma^{2}-\sigma_{w}^{2}(T)}$$

**Figure 3:**
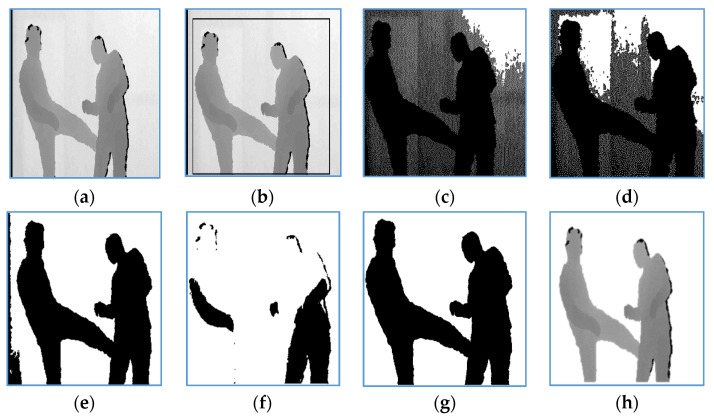
Depth silhouette segmentation of kicking interaction from SBU dataset: (**a**) original image; (**b**) initial ROI; (**c**) binary image at *T* = 0.25, (**d**) binary image at *T* = 0.22 (**e**) binary image at *T* = 0.20, (**f**) binary image at *T* = 0.13, (**g**) segmented binary silhouette at *T* = 0.19; (**h**) segmented depth silhouette.

In this way, depth silhouettes are separated from their background. Figure 3 demonstrates an example of the depth silhouette segmentation of kicking interaction from the SBU dataset.

### 3.3. Hybrid Feature Extraction 

After the extraction of silhouettes from complex backgrounds, we proposed a novel hybrid feature extraction method. This method is a fusion of key-body point features and full silhouette features. Spatio–temporal and angular–geometric features are based on key-body points while motion-orthogonal HOG and energy-based features are based on full silhouettes. These four novel features are extracted and discussed in sub-sections below.

#### 3.3.1. Spatio–Temporal Feature

Spatial features give information regarding changes with respect to space, location or position [68]. For spatial features, we measured intensity changes along the curve points of the body using the 8 Freeman chain code algorithm. These features are extracted along the boundary of the human silhouette because a small change in the position of a human silhouette results in changes in the curves of silhouette. So, in order to extract spatial features, we first identified the boundaries of the two human silhouettes involved in the interaction. Then, all the curve points along the human contour of both silhouettes were identified and represented using the 8 Freeman chain code. If we suppose that all the points along the boundary *b* are represented by *n* points, then curve points $C_{b}$ along the boundary are found from starting point $C_{0}$ to *n −* 1 as $C_{b}=\left\{C_{0},C_{1}\ldots \ldots .C_{n-1}\right\}$.

**Figure 4:**
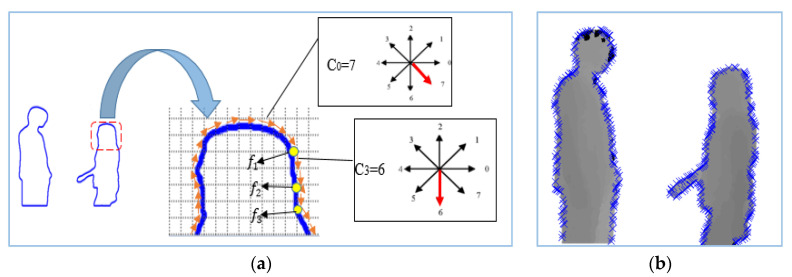
Spatial feature extraction: (**a**) method to find a spatial feature point; (**b**) depth silhouette of approaching interaction with marked feature points.

Moreover, we start to find a feature point from curve $C_{0}$ and move in a clockwise direction along with the boundary until we observe a change in direction from $C_{0}$. Suppose that $C_{0}$ is the current curve point and $C_{1}$ is the next point and if the direction of both $C_{0}$ and $C_{1}$ is the same then we will move to next curve point $C_{2}$. If the directions of both $C_{0}$ and $C_{1}$ are not the same, then we will consider $C_{1}$ as a feature point *f* (see Figure 4a). So, we will consider a curve point to be a feature point *f* if the difference between current curve point and the next curve point is not equal to 0. In this way, spatial feature finds almost all the parts of body of a human silhouette (see Figure 4b). Figure 4 demonstrates the overall procedure to find the feature points using the 8 Freeman chain code.

**Figure 5:**
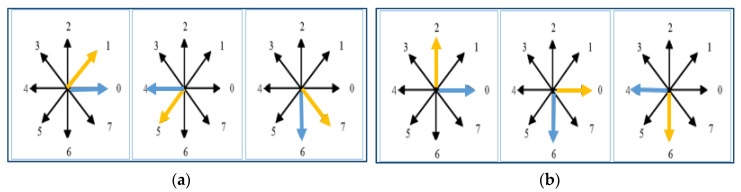
Cases of spatial feature point extraction: (**a**) three cases of 45° change in direction; (**b**) three cases of 90° change in direction.

In order to find a feature point, we have taken eight cases of 45° and four cases of 90° to find changes in the direction of each curve point. Figure 5 describes a few cases of 45° and 90° change in direction in which yellow arrows show the current curve point direction while the blue arrows show the subsequent curve point direction.

Temporal features give information about changes with respect to time. In order to extract temporal features, critical displacement measurements between eight key-body points [69,70] are considered. Initially, our system tracked eight key-body points (head, left shoulder, right shoulder, left arm, right arm, left foot, right foot and torso) on detected RGB and depth silhouettes. These silhouettes were converted to binary and then the outer boundaries of silhouettes were identified. Then, different positions, such as the topmost, right most, left most, bottom left most, bottom right most and center point of a human silhouette, are identified. Algorithm 1 presents the overall procedure used for the key-body point detection of human silhouettes.
**Algorithm 1** Detection of key-body points human silhouette**Input:** S: Segmented Human Silhouettes**Output:** 8 key-body points as Head, Left-shoulder, Right-Shoulder, Left-arm, Right-arm, Left-foot, Right-foot, Torso.B1 = boundary of left silhouette, B2 = boundary of right silhouette, H = height of silhouette, W = width of silhouette**%** Extract boundaries of both silhouettes **%***B = binarize(S);**BW = bwboundaries(B);**Object = detectobject(B,Boundingbox, Area)***%** search boundaries of both silhouettes for outermost pixels **%****for***i* = 1 to *B1***for***j* = 1 to *B2*Search (B1;B2)*Top_Pixel = [x, y_max];**Left_pixel = [x_min,y];**Rightl_pixel = [x_max,y];**Bottom_left_pixel = [x_min,y_min];**Bottom_right_pixel = [x_max,y_min];***end****end****%** detect top, bottom, left and right region of both silhouettes **%****%** Repeat for both silhouettes **%***[rows, cols] = size(object)**Top_half = floor(rows/2)**bottom_half = rows-Head_region**Head_region = floor(Top_half/3)**Torso_region = floor(Top_half-Head_region)*% identifying head region in top half of silhouette%*Head = Top_Pixel(Head_Region)**Left_Shoulder = Bottom_left_pixel(Head_Region)**Right_Shoulder = Bottom_right_pixel(Head_Region)*% identifying Torse, left arm and right arm%*X_t_ = (W/2)**Y_t_ = (H/2)**Torso = (X_t_, Y_t_)**Left_arm = Left_Pixel(Torso_region)**Right_arm = Right_Pixel(Torso_region)**%* identifying left foot and right arm %*Left_foot = Bottom_left_pixel(bottom_half)**Right_foot = Bottom_right_pixel(bottom_half)***return** Head, Left-shoulder, Right-Shoulder, Left-arm, Right-arm, Left-foot, Right-foot, Torso.

**Figure 6:**
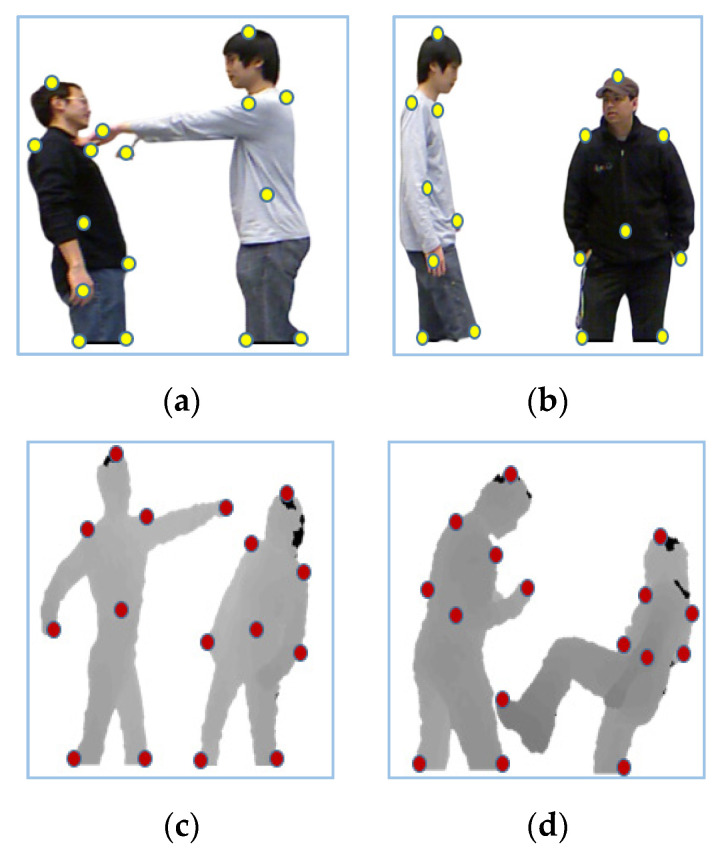
Key-body points on RGB and depth frames of: (**a**) pushing; (**b**) approaching; (**c**) punching; (**d**) kicking interaction of SBU dataset.

After identifying key-body points, position displacement measurement between all key-body points of the first person’s silhouette (silhouette of person on left side) and all key-body points of the second person’s silhouette (silhouette of person on right side) are measured as shown in Equation (5):

(5) D(p,q)=(px−qx)^2+(py−qy)^2

where *D*(*p*, *q*) is Euclidian distance, $p_{x}$ and $p_{y}$ are *x*, *y* coordinates of the key body points of the first person’s silhouette and $q_{x}$ and $q_{y}$ are *x*, *y* are coordinates of the second person’s silhouette. As a person moves or performs any interaction, the distance between these key-body points may increase or decrease in values. Key-body points for both RGB and depth images are shown in Figure 6.

#### 3.3.2. Angular–Geometric Features

An angular–geometric feature is a shape-based entity defined as a key-body point feature. In order to extract angular and geometric features, seven extreme body points (head, left shoulder, right shoulder, left arm, right arm, left foot, right foot) are first identified. Then, three geometric shapes—i.e., pentagon, quadrilateral and triangle—are made by joining these extreme points. In angular features, we measure changes in angular values between extreme point positions in consecutive frames. Two types of geometric shapes are made by joining these extreme points, such as: inter-silhouette shapes and intra-silhouette shapes. Table 1 shows a detailed overview of a number of inter-silhouette and intra-silhouette shapes and angles that are made by joining the extreme body points of silhouettes.

Inter-silhouette shapes are made within each silhouette. These are geometric shapes made by connecting the extreme points of each silhouette individually. Intra-silhouette shapes are made between two silhouettes by connecting the extreme points of one silhouette with the extreme points of the second silhouette within each frame. After the completion of both types of geometric shapes, the inverse cosine angle is measured between all these shapes, as shown in Equation (6):

(6) $$\theta =\cos^{-1}\frac{u.v}{\left|u\right|\left|v\right|}$$

where *u* and *v* are the two vectors in which the angle is measured. After measuring the angles, the shape’s areas of all the inter-silhouette and intra-silhouette triangles are calculated. The area of the triangle is measured through Equation (7):

(7) $$A_{t}=\sqrt{S(S-a)(S-b)(S-c)}$$

where *a, b* and *c* are three sides of the triangle in which vectors are joined together—i.e., three extreme points to make a triangle—and *S* is the semi-perimeter of a triangle—i.e., half the length of the triangle’s perimeter.

With the movement of each extreme point during interaction, the area of each geometric shape may increase or decrease. So, angular and geometric features measure changes in the angles as well as changes in the area of each shape between consecutive frames. The rate of change for the angles and the area are more evident in interactions like fight and kick, because they involve rapid movements of the extreme points during interaction as compared to approaching and departing interactions that include less pronounced movements of the extreme points.

#### 3.3.3. Motion-Orthogonal Histogram of Oriented Gradient (MO-HOG)

**Figure 7:**
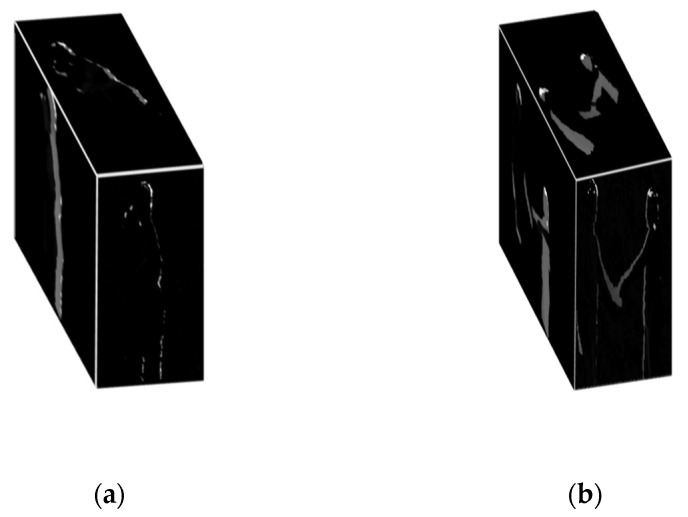

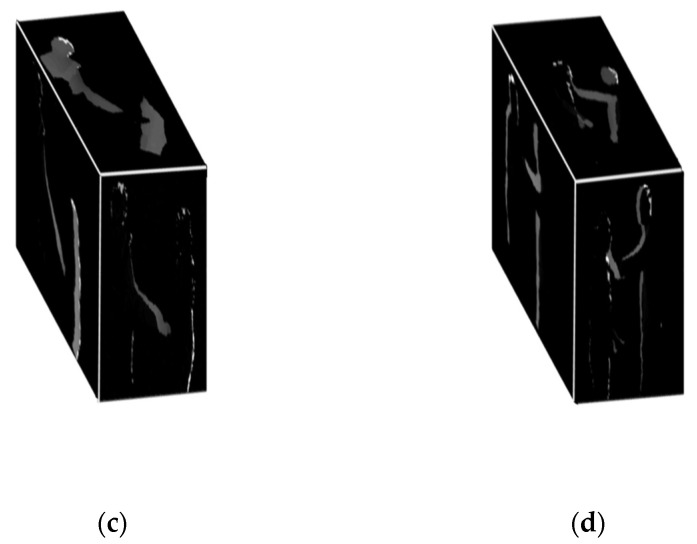
Orthogonal projection of 3D views of DS for: (**a**) hugging, (**b**) shaking hands, (**c**) kicking and (**d**) pushing interactions.

MO-HOG is a motion-based feature applied over full silhouettes. It was observed that, in most of the interactions, the postures of both humans’ silhouettes remain the same. For example, in approaching, departing, pushing and talking, the front views of both humans look like they are standing with only slight movements. Interactions like exchanging object and shaking hands are hardly distinguishable from each other. Punching and pushing interactions also have similar body movements. Therefore, our system proposed a novel multi-views approach including front, side and top views of both RGB as well as depth silhouettes by using a 3D Cartesian planes approach [71]. In order to incorporate motion data, we created RGB and depth differential silhouettes (DS) by taking differences between top *t*, front *f* and side *s* views of two consecutive frames as defined by Equation (8):

(8) $$DS{\left(Fc\right)}_{f,s,t}=|Fc_{f,s,t}-Fp_{f,s,t}|$$

where *Fc* is current frame and *Fp* is previous frame. After taking DS of multi-views of silhouettes, they are projected as 3-dimensional (3D) Cartesian planes in the form of orthogonal shapes, as shown in Figure 7.

**Figure 8:**
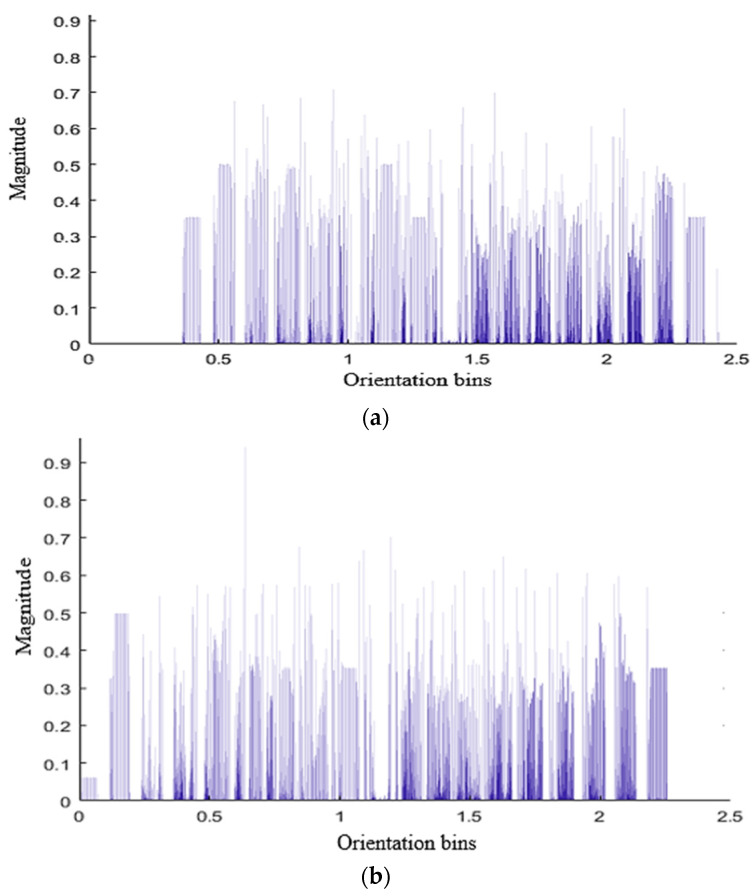
A bar graph showing HOG feature vector of (**a**) hugging and (**b**) kicking interactions.

These multi-view DS are fed into HOG to extract orientation features [72]. It calculates magnitude and gradient by dividing the image into 8 × 8 cells which are stored in a 9-bin histogram. A bar graph shows the magnitude and orientation bins of different interactions in Figure 8.

#### 3.3.4. Energy-Based Features

**Figure 9:**
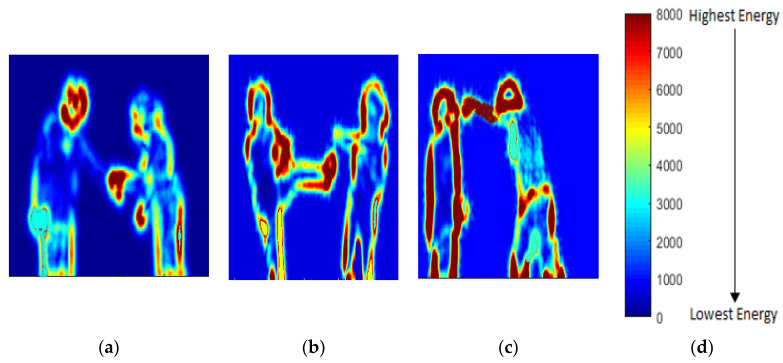
Energy features applied over (**a**) shaking hands; (**b**) kicking; (**c**) punching interactions of SBU dataset; (**d**) color bar showing energy range.

In energy-based features, the movements of human body parts are captured in the form of Energy Maps (EMs). EMs distribute the energy matrix between a set of [0–8000] indexes values over the detected silhouette. After energy distribution, a threshold-based technique is used in which only higher energy index values that are greater than a specified threshold are extracted into a 1D vector. Energy distribution is represented by Equation (9):

(9) $$ER(v)=\sum_{0}^{N}\ln R(N)$$

where *ER*(*v*) is 1D energy vector, *N* is the index number, and In *R* is the RGB values of *N*. Energy distributions over some interactions of the SBU dataset are shown in Figure 9.

In Figure 9a, most of the energy is distributed in the region of hands. In Figure 9b, most of the energy is distributed in the left foot and the left shoulder because, when a person kicks, the upper body moves a little backward. Lastly, in Figure 9c, when the right silhouette starts punching, it moves forward, while the left silhouette moves backward as a reaction. Thus, energy distribution occurs at hands and the head of the right silhouette and around the whole body of the left silhouette. These energy maps show the energy of the body parts that are involved in the interaction in a red or a darker color. Meanwhile, those parts of the human body that are not involved during the interaction are in blue or lighter colors. Algorithm 2 explains the hybrid feature extraction algorithm.
**Algorithm 2** Hybrid feature extraction**Input:** N: Segmented Silhouettes frames of RGB and RGB-D images**Output:** Hybrid feature vectors(*k*_1_, *k*_2_, *k*_3_, …, *k_n_*)% initiating feature vector matrix %Hybrid Feature-vectors ← []Vectorsize ← GetVectorsize ()% for loop on segmented silhouettes frames of all interaction classes %**for***i* = 1:N vectors_interactions ← Getvectors(interactios)     % extracting spatio-tempora, MO-HOG, angular-geometric and energy features %*Spatio-temporal* ← ExtractSpatioTemporalFeatures(vectors_interactions)*MO-HOG* ← ExtractOrthogonalHOGFeatures(vectors_interactions)*Angular-Geometric* ← ExtractAngularGeometricFeatures(vectors_interactions)*Energy* ← ExtractEnergyFeatures (vectors_interactions)*Feature-vectors* ← GetFeatureVectors (spatio-tempora, MO-HOG,Angular-Geometric, Energy)Hybrid Feature-vectors.append (Feature-vectors)**end***Hybrid Feature-vectors* ← Normalize (Hybrid Feature-vectors)**return** Hybrid Feature-vectors(*k*_1_, *k*_2_, *k_3_*………*k_n_*)

### 3.4. Codebook Generation

After extracting the hybrid features of both RGB and depth images, feature descriptors of all image sequences are combined to form a matrix representation. Such a matrix representation is so assorted and complex that there is a need to represent it in a sorted and simpler way. Therefore, we applied Fisher vector encoding (FVC), based on GMM for codebook generation [73]. Initially, we applied GMM to compute the mean and covariance of each class, separately [74]. Based on computed values, clusters of each class are generated. Thus, the probability density function (pdf) of the cluster of the *d* dimensional vector *X* is defined by through Equation (10):

(10) $$p(X;\theta )=\sum_{k=1}^{K}w_{k}N(X|\mu_{K},\sum_{K})$$

where *θ* = {$w_{k}$, $\mu_{k}$, $\Sigma_{k}$ *| k =* 1, 2, …, *K*}, $w_{k}$ is the weight of *k*th Gaussian component, *K* is the total number of clusters, the mean value is represented through $\mu_{k}$, the covariance matrix is given by $\Sigma_{k}$ and *N* represents the distribution of *d* dimensional Gaussian. In addition, Expectation Maximization estimates the maximum likelihood of parameters of GMM. During expectation maximization soft assignment of vectors $x_{t}$ to their belonging Gaussian cluster *k* is learned through Equation (11):

(11) $$q_{t}(k)=\frac{w_{k}N(x_{t};\mu_{k},\sum {}_{k})}{\sum_{j=1}^{K}w_{j}N(x_{t};\mu_{j},\sum {}_{j})}$$

After applying GMM, FVC is performed on feature descriptors. *X =* {$x_{t}$, *t =* 1, 2, …, *T*} is a given feature set, while Gradient of log likelihood $\nabla_{\theta }$ of *X* having GMM parameters *θ* is given through Equation (12):

(12) $$F_{X}=\frac{1}{T}\nabla_{\theta }\log p(X;\theta )$$

where $F_{X}$ is feature vector. Now, the gradient vector is computed with respect to each mean $\mu_{k}$ and covariance $\sigma_{k}$ defined by Equations (13) and (14), respectively.

(13) $$\mu_{k}=\frac{1}{T\sqrt{w_{k}}}\sum_{t=1}^{T}q_{t}(k)\frac{x_{t}-\mu_{k}}{\sigma_{k}}$$

**Figure 10:**
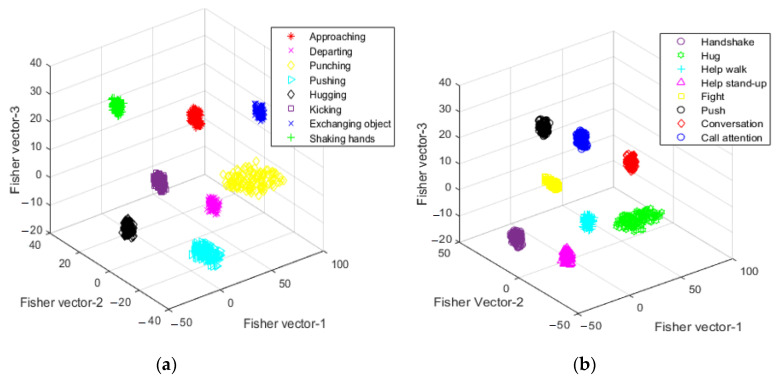
Three-dimensional clusters of FVC over: (**a**) SBU dataset and (**b**) UoL dataset.

Finally, all computed gradient vectors $\mu_{k}$ and $v_{k}$ for *K* components are combined to form *D*-dimensional final encoded feature vectors of dimensions 2KD. Hence, the Fisher vector reduces the intra-cluster gap and increases the inter-cluster gap, which gives a more precise discrimination of each cluster. Figure 10 demonstrates clusters formed by each interaction as a result of FVC over SBU and UoL 3D datasets.

### 3.5. Cross Entropy Optimization

In order to reduce the complexity of fisher encoded vectors, a cross entropy technique is implemented [75]. In cross entropy, initially, a sample of a specified size is generated from the fisher encoded vectors of each interaction class and an objective function is applied to that sample [76]. Then, more samples are extracted from encoded vectors and their objective functions are compared. This process continues until maximum numbers of iterations are reached or the best sample is obtained. The best sample of descriptors would represent an interaction class with the optimal set of descriptors. So, several iterations are performed until an optimal sample is generated. Cross entropy is measured among two probability distributions with samples *p* and *q*, and this is represented through Equation (15):

(14) $$H(p,q)=-\sum_{x}^{X}p_{x}\log q_{x}$$

where $p_{x}$ and $q_{x}$ are probabilities of event *x* (i.e., $p_{x}$ is an actual or true value of probability and $q_{x}$ is the predicted value). Meanwhile, Kullback–Leibler divergence *D* is calculated between true probability and predicted probability by Equation (16):

(15) $$D_{KL}(p|q)=\sum_{i=1}^{N}p(x_{i})\cdot (\log p(x_{i})-\log q(x_{i}))$$

**Figure 11:**
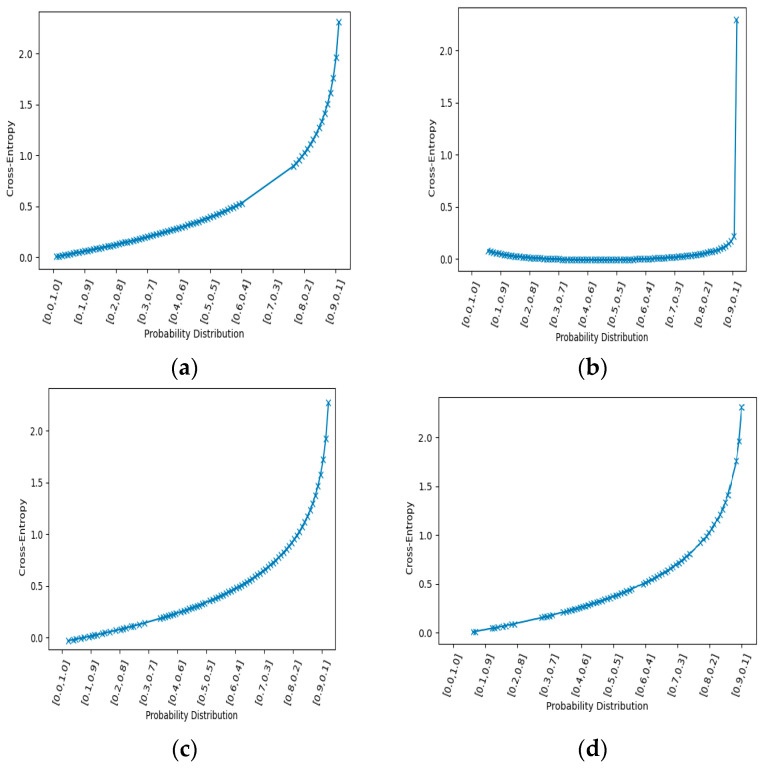

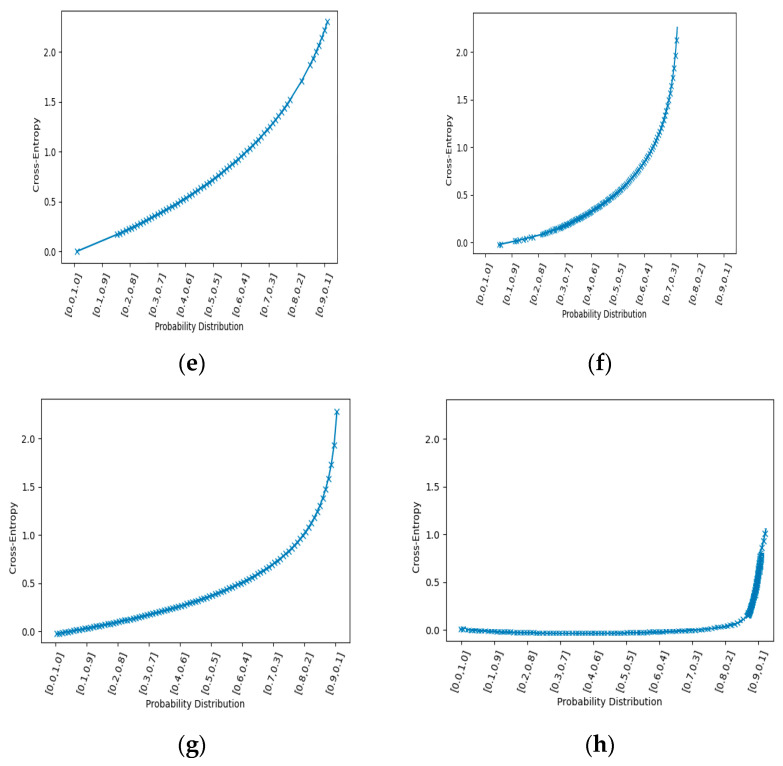
Cross entropy between probability distributions of interaction classes of SBU dataset: (**a**) approaching; (**b**) departing; (**c**) exchanging object; (**d**) punching; (**f**) kicking; (**g**) hugging; (**h**) shaking hands.

In this way, the difference between the true and the predicted probability of a given sample is calculated. Cross entropy between the predicted and true probability distribution of each class of SBU dataset is shown in Figure 11.

### 3.6. Classification Via MEMM

**Figure 12:**
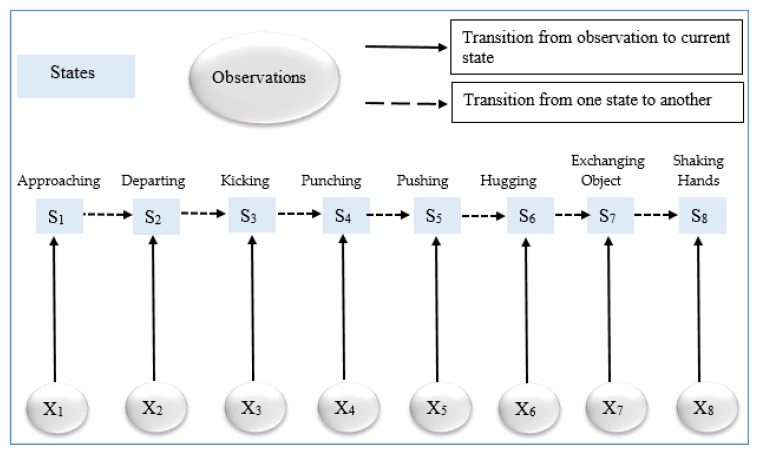
Overall flow of MEMM recognizer engine at different interaction classes of SBU Kinect interaction dataset.

After getting an optimized representation of the vectors, they are fed into a maximum entropy- based classifier in order to determine the different interaction classes (see Algorithm 3). MEMM is a combination of both the HMM and the maximum entropy model [77]. It is a discriminative model where a conditional probability is used to predict the interaction class. Such conditional probability is represented as *P (S|S’, X)*. Each transition between state and observation in the MEMM is given through a log linear model, which is represented in Equation (16):

(16) $$P_{S{}^{\prime }},(S|X)=\frac{1}{Z(X,S{}^{\prime })}\exp(\sum_{k}\lambda_{k}f_{k}(X,S))$$

where *S* is the current state, *S*’ is the next state, *X* is an observation, $f_{k}$ is a feature function of *X* and possible *S*’, *Z*(*X*,*S*’) is a normalization factor that ensures the matrix sum and $\lambda_{k}$ is the weight to be learned and is associated with feature $f_{k}$. According to the above observations, it is clear that MEMM is not only dependent on current observations but also on the previously predicted interaction. Figure 12 shows the overall procedure of the MEMM over different interaction classes of SBU Kinect interaction dataset.
**Algorithm 3** Vector Optimization and Interactions Classification**Input:**: Hybrid feature vectors *(V_1_,V_2_,……….V_N_)*GMM parameters *θ* = *{*$\pi_{k}$*,*
$\mu_{k}$, $\Sigma_{k}$
*where k = 1,2,…..K}***Output:** Recognized Interaction I = *{I_1_*, *I_2_*, *I_3_*,……*I_n_}*                % Fisher Vector Encoding %FisherVector ← []**for***V* = 1:V_N_ where V_N_ is total no. of vectorsdeviance_mean← ComputeGradiantVector($\pi_{k}$)deviance_covariance← ComputeGradiantVector($\Sigma_{k}$)     %concatenate deviance w.rt mean and covariance matrix of all vectors in N%FisherVectors←Concatenate(deviance_mean, deviance_stand_dev)FisherVectors←FisherVector.append(FisherVector)**end**              % Cross Entropy Optimization %Best_Sample← []**while** t < T where t is current iteration and T is total number of iterations**for***i* = 0:S_N_ where S_N_ is maximum no. of Samples**Sam**← ExtractSamples(FisherVectors)**end**ComputeSamplePerformance (Sam)ComparePerformance (Sam, Best_Sample)SortSamplebyPerformance (Sam)Selected_Sample←ChooseBestSample (Sam)Best_Sample← (Selected_Sample)**end****return** optimized vector            % Classifying interactions via MEMM %Recognized interaction←[]Initialize State S = {X_1_, X_2_……….X_T_} where T = total no. of statesObservations = {O_1_,O_2_……O_N_} where O_N_ is total no. of observations           % Suppose a random state to be a current state %X_t_ ← CurrentState**while** state            % suppose S_f_ state to be determining state %S_f_ ← StatetoFind              % ComputeCoditionalprobability %S_f_ ← ComputeStatetoFind(S_f_ | S_f-1_, O_t_)state ← S_f_
X_t_ ← state**end****return** state as Recognized interaction *{I_1_*, *I_2_*, *I_3_,……I_n_}*


## 4. Experimental Setting and Results

In this section, we report training/testing experimentation results using the *n*-fold cross validation method over three publicly available benchmarks datasets. SBU Kinect interaction and UoL 3D datasets include both RGB and depth image sequences while UT interaction dataset consists of RGB data only. Furthermore, complete descriptions of each dataset are given in this section. The proposed model is evaluated on the basis of various performance parameters—i.e., computation time, recognition accuracy, precision, recall, F1 Score, number of states and number of observations. Discussion about various well-known classifiers and comparison of the proposed HIR with other statistically known state-of-the-art HIR methods is also given in this section.

### 4.1. Datasets Description

#### 4.1.1. SBU Kinect Interaction Dataset

**Figure 13:**
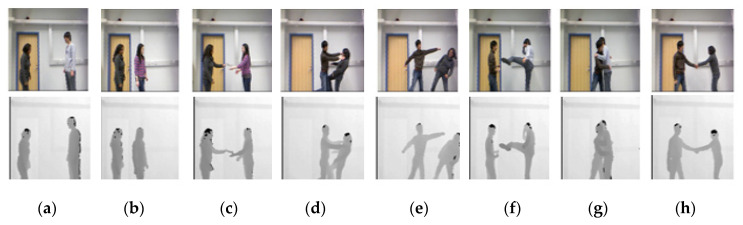
RGB and depth snapshots of interaction classes of SBU dataset. (**a**) Approaching; (**b**) departing; (**c**) exchanging object; (**d**) pushing; (**e**) punching; (**f**) kicking; (**g**) hugging; (**h**) shaking hands.

The SBU Kinect interaction dataset [78] consists of RGB, depth and skeletal information for the two-person performing interactions collected by Microsoft Kinect sensors in an indoor environment. Eight types of interactions including Approaching, Departing, Kicking, Punching, Pushing, Shaking Hands, Exchanging Object and Hugging are performed. The overall dataset is really challenging to interpret due to the similarity or closer proximity of movements in the different interaction classes. The sizes of both RGB and depth images are 649 × 480. Additionally, the dataset has a total of 21 folders, where each folder consists of all eight interaction classes performed by a different combination of seven actors. The ground truth labels of each interaction class are also provided. Videos are segmented at the rate of 15 frames per second (fps). Figure 13 shows some examples of human interaction classes of the SBU dataset.

#### 4.1.2. UoL 3D Dataset

**Figure 14:**
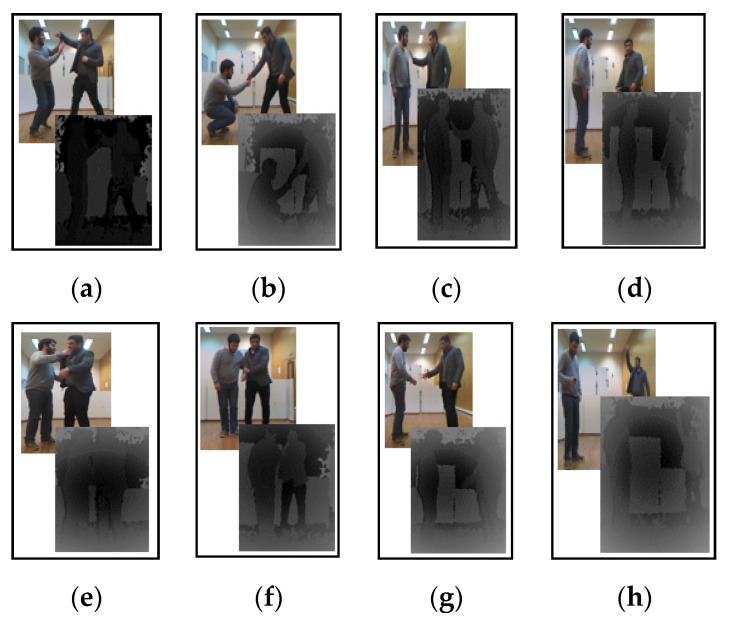
RGB and depth snapshots of interaction classes of UoL 3D dataset: (**a**) fight; (**b**) help stand-up; (**c**) push; (**d**) conversation; (**e**) hug; (**f**) help walk; (**g**) handshake; (**h**) call attention.

In the UoL 3D dataset, there is a combination of three types of interaction, such as casual daily life, harmful and assisted living interactions [79]. Included are interactions, such as handshake, hug, help walk, help stand-up, fight, push, conversation and call attention, performed by four males and two females. In addition, RGB, depth and skeletal information for each interaction is captured through the Kinect 2 sensor. Each folder has 24-bit RGB images, 8-bit and 16-bit resolution depth images of both 8-bit and 16-bit resolution and the skeletal information has 15 joints. There are ten different sessions of eight interactions performed by two subjects (in pairs), which are recorded in an indoor environment for period of 40–60 repetitions. This is a very challenging dataset and consists of over 120,000 data frames. Some snapshots of interactions of this dataset are shown in Figure 14.

#### 4.1.3. UT Interaction Dataset

**Figure 15:**
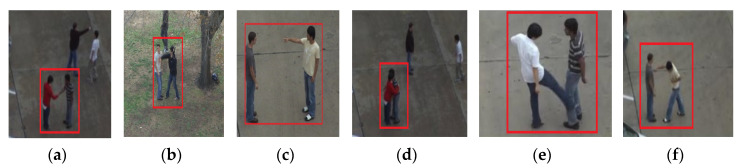
Few examples of interaction classes of UT-Interaction dataset. (**a**) Shake hands; (**b**) push; (**c**) point; (**d**) hug; (**e**) kick; (**f**) punch.

The UT interaction dataset [80] consists of only RGB data. It has six interaction classes: point, push, shake hands, hug, kick and punch performed, by several participants with different appearances. This dataset is divided into two sets, named as: UT-Interaction Set 1 and UT-Interaction Set 2. The environment of Set 1 is a parking lot and the environment of Set 2 is a windy lawn. Video is captured with a resolution of 720 × 480 at 30 fps. There are 20 videos per interaction providing a total of 120 videos of six interactions. Figure 15 demonstrates some examples of interaction classes for UT-Interaction dataset.

### 4.2. Performance Parameters and Evaluation

In order to validate the methodology of the proposed HIR system, four different types of experiments with various performance parameters—i.e., recognition accuracy, precision, recall, F-score, computational time and comparison with state-of-the art methods—were performed. Details and observations for each experiment are discussed in the sub-sections.

#### 4.2.1. First Experiment

In the first experiment, optimized feature vectors are subjected to MEMM in order to evaluate the average accuracy of the proposed system. We used the *n*-fold cross validation method for training/testing over three benchmark datasets. Table 2 and Table 3 show the accuracy of the interactions of SBU and UoL datasets in the form of a confusion matrix. Similarly, recognition accuracies of UT-Interaction Set 1 and Set 2 are shown in Table 4 and Table 5, respectively. While, the mean accuracy of the SBU dataset is 91.25%, the accuracy of UoL is 90.4% and the combined accuracy of the UT-Interaction Set 1 and Set 2 is 87.4%.

From the experimental results, it is observed that our hybrid features methodology, along with cross entropy optimization and the MEMM, can clearly recognize human interactions better. However, some confusion is observed between pairs of similar interactions, such as shaking hands and exchanging object, and punching and pushing interactions, in the SBU dataset. In the UoL dataset, confusion is observed between handshake and help stand-up interactions. Such confusion is due to the similarity in body movements involved in these interactions. In the UT-interaction dataset, there is confusion between shaking hands and point, and push and punch interactions due to similarities of these interactions. In addition, it is also observed that when combinations of RGB and depth vectors were fed into the MEMM, we achieved better recognition rates compared to RGB alone. The recognition rate of the RGB dataset i.e., UT interaction (87.4%) is less than those of the SBU and the UoL datasets, which are 91.25% and 90.4%, respectively. Thus, incorporating depth information results causes improvements in accuracy rate.

#### 4.2.2. Second Experiment

In the second experiment, precision, recall and F1 Score for each interaction class of three datasets are evaluated, as shown in Table 6.

It is observed that, in the SBU dataset, the Approaching interaction has the least precise rate of 88% and it also has a highest rate of false positive. This is because many periodic actions of many interactions such as departing, shaking hands and exchanging object are similar to the approaching interaction. On the other hand, the kicking interaction gives the most precise results with a less false positive ratio of 3%. In the UoL dataset, Hug interaction gives the most precise result of 95% because the periodic actions performed during the Hug interaction are different from the other interactions of this dataset. Handshake and conversation interactions have the highest false positive ratios of 13% and 14%, respectively, because body movements of silhouettes during these two interactions are similar to many other interactions. Overall, if we compare three datasets, the precision recall and F1 score ratios of both sets of the UT Interaction dataset are less as compared to the SBU and UoL datasets.

#### 4.2.3. Third Experiment

In the third experiment, nine sub-experiments for each dataset were performed. In this experiment, different combinations of the two parameters (i.e., number of states and observations) were used to evaluate the performance of MEMM. As a result, comparisons are made in terms of time complexity and recognition accuracy. During MEMM, each transition not only depends on the current state but also on the previous state. Therefore, increasing the number of states and observations affects the performance rate of HIR. Table 7, Table 8 and Table 9 show a comparison of number of states and observations for time complexity and recognition accuracy over the SBU, UoL 3D and UT-Interaction datasets.

In Table 7, by using four states and changing the number of observations from 10 to 30, computational time and recognition accuracy were gradually increased. These experiments are repeated for five and six states. Similarly, Table 8 used 4–6 states and received significant results for computational time and recognition accuracy at 15 to 35 numbers of observations. Table 9 presents the results of these experiments on Set 1 of the UT-Interaction dataset, respectively.

It is concluded from the third experiment that reducing the number of states to two reduces recognition accuracy and computational time. On the other hand, increasing the number of states to six results in increased computational time with no change in accuracy. However, similar patterns of observations are noticed in Table 7, Table 8 and Table 9 (i.e., increasing the number of states and observations results in increased computational time and accuracy as well).

#### 4.2.4. Fourth Experiment

In the fourth experiment, we compared our proposed system in two parts. In the first part, a hybrid descriptor-based MEMM classifier is compared with other commonly used classifiers. In the second part, the proposed system is compared with other statistically well-known state-of-the-art HIR systems.

**Figure 16:**
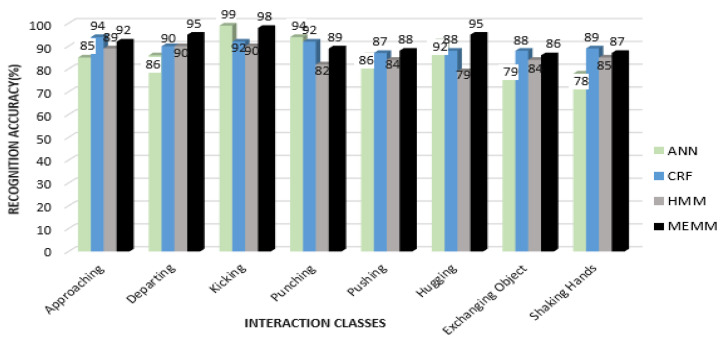
Comparison of other classifiers with MEMM over interaction classes of SBU dataset.

In the first part, quantized features vectors are given to most commonly used classifiers—i.e., ANN, HMM and Conditional Random Field (CRF)—and compared with MEMM to find the HIR accuracy rates for the interactions of each dataset. Figure 16 shows a comparison of recognition accuracies for each interaction class of the SBU dataset using all four classifiers.

**Figure 17:**
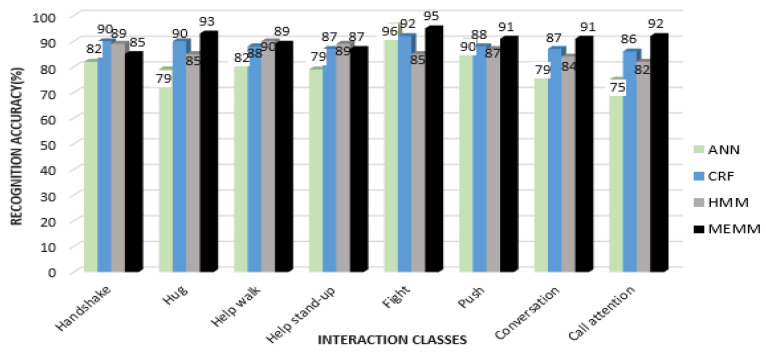
Comparison of other classifiers with MEMM over interaction classes of UoL dataset.

From Figure 16, it can be seen that the mean recognition accuracy for ANN is 87.3%, CRF is 90%, HMM is 85.3% and MEMM is 91.25%. It is observed that, in some interactions, such as exchanging object and shaking hands, CRF performed better than MEMM. Additionally, ANN performed better in a few interactions, such as kicking and punching. Overall accuracy using the MEMM was higher than for other classifiers. Figure 17 shows the comparison of recognition accuracies for each interaction class using the UoL dataset.

**Figure 18:**
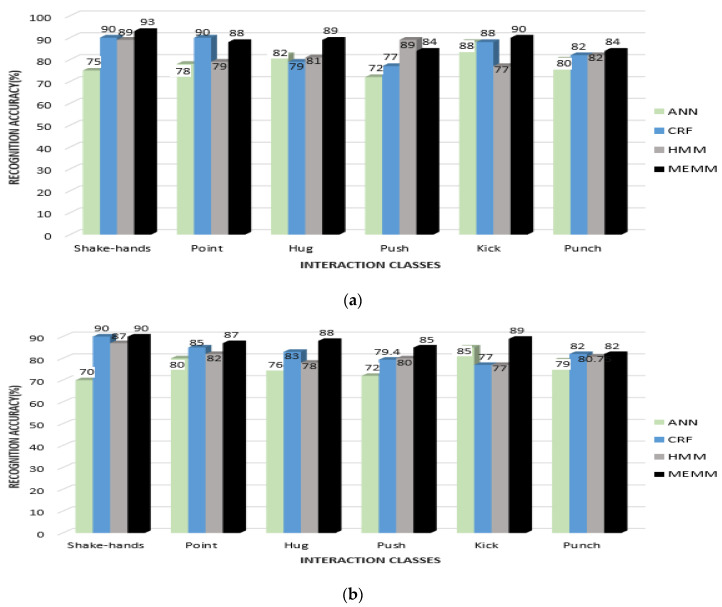
Comparison of other classifiers with MEMM over interaction classes of UT-Interaction: (**a**) Set 1 and (**b**) Set 2.

From Figure 17, it is shown that the mean recognition accuracy of ANN is 82.75%, CRF is 88.5%, HMM is 86.37% and MEMM is 90.4%, using the UoL dataset. It is observed that some interactions, such as fight in the case of ANN, handshake in the case of CRF and help walk in the case of HMM, achieved better recognition accuracy than the MEMM. However, the overall recognition rate was still higher with the MEMM. Figure 18 shows the comparison of four classifiers over interaction classes of the UT-Interaction Set 1 and Set 2.

From Figure 18a,b, it is observed that the mean recognition accuracy rates of Set 1 and of Set 2 for the UT-interaction dataset are less than the depth datasets. The mean accuracies of Set 1 of the UT Interaction dataset are 79.16% with ANN, 84.3% with CRF, 82.8% with HMM and 88% with the MEMM classifier. Mean accuracies are further reduced with Set 2 for the UT interaction dataset due to the cluttered background of a windy lawn. The mean accuracy for ANN is 77%, CRF is 82.7%, HMM is 80.7% and MEMM is 86.8%. Meanwhile, it is observed that patterns of recognition accuracies for Set 1 and for Set 2 are similar to those of the UoL and of the SBU datasets and that the MEMM has the highest accuracy rate while ANN has the lowest. Accuracy rates for the MEMM and CRF are comparable. Moreover, CRF, HMM and MEMM performed better in most of the interactions classes except for the fight interaction, where ANN has better or nearly similar recognition rate. However, overall MEMM has best recognition rates. Thus, it is concluded that MEMM based performance is best for HIR.

In second part of this experiment, the proposed HIR system is compared with other statistically well-known state-of-the-art systems. Table 10 presents a comparison of results for the SBU, UoL and UT interaction datasets, respectively.

## 5. Discussion

A unique HIR system is proposed in this research work. Four unique features are extracted from both RGB and depth silhouettes. The efficiency of the proposed model is proved through four types of experiments. However, certain challenges were faced during this research work. In the silhouette detection phase of the RGB frames, a connected components algorithm was used to identify connected objects. However, this algorithm does not give the best results as it confuses white or light color clothes of individual with the white wall background. Therefore, in order to tackle this problem, we applied a human skin detection algorithm as well as a pre-specified measurements ratio (i.e., the height and width) of the human performers. This ratio is compared with the height and width ratio of bounding boxes of connected components. Again, the specific height and width ratio of human causes the failure of silhouette detection due to frequent changes in scaling values of human posture.

## 6. Conclusions and Future Work

In this paper, we have proposed a novel HIR system to recognize human interactions using both RGB and depth environmental settings. The main accomplishments of this research work are: (1) we achieved adequate silhouette segmentation; (2) identification of key human body parts; (3) extraction of four novel features—i.e., spatio-temporal, MO-HOG, angular-geometric and energy based features; (4) cross-entropy optimization and recognition of each interaction via MEMM. In the first phase, both RGB and depth silhouettes are identified separately. For RGB silhouette segmentation, various skin colors, connected components and binary thresholding methods are applied to separate humans from their background. After the extraction of silhouettes, all spatio–temporal features are extracted. In these features the displacement between key body points is identified via Euclidean distance. In angular–geometric features, various geometrical shapes are made by connecting the extreme points of silhouettes. The angles of these shapes are then measured in each interaction class. After that, MO-HOG features are extracted, in which differential silhouettes are projected from three different views and then HOG is applied. Finally, unique energy features are extracted from each interaction class. A Hybrid of these feature descriptors results in very complex vector representation. In order to reduce the complexity of the feature descriptors, a GMM based FVC is applied and then cross entropy optimization is performed.

During experimental testing, four different types of experiments were conducted on three benchmark datasets in order to validate the performance of the proposed system. In the first experiment, recognition accuracies for the interaction classes of each dataset were measured. In the second experiment, F1 scores, precision and the recall of each interaction class were measured and compared. In the third experiment, computation time and accuracy were measured by changing the number of states and observations of the MEMM classifier. Finally, in the fourth experiment, recognition accuracies for the interaction classes of each dataset were measured via the most commonly used classifiers—i.e., ANN, HMM and CRF—and compared with MEMM. Results showed better performance, with an average recognition rate of 87.4% for UT-Interaction, 90.4% for UoL and 91.25% for SBU datasets. Results of these experiments validated the efficacy of the proposed system. The proposed system is applicable to various real-life scenarios, such as security monitoring, smart home, healthcare and content-based video indexing and retrieval, etc.

In the future, we plan to implement the proposed method in a group of human interactions as well as human–object interactions. We will also use entropy-based features. We will also work on more challenging datasets.

## Figures and Tables

**Table 1:** Properties of inter-silhouette and intra silhouette geometrical shapes.

Type of Geometrical Shape	Connected Extreme Points	No. of Angles	Diagrammatical Representation
Inter-Silhouette Triangle	H1 ^1^ + RS1 ^2^ + LS1 ^3^	18	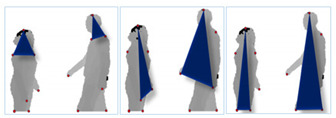
	H1 + RA1 ^4^ + LA1 ^5^		
	H1 + RF1 ^6^ + LF1 ^7^		
	H2 ^8^ + RS2 ^9^ + LS2 ^10^		
	H2 + RA2 ^11^ + LA2 ^12^		
	H2 + RF2 ^13^ + LF2 ^14^		
Inter-Silhouette Quadrangular	RS1 + LS1 + RA1 + LA1	24	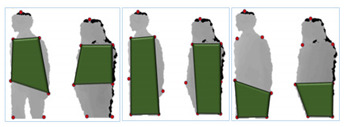
	RA1 + LA1 + RF1 + LF1		
	RS1 + LS1 + RF1 + LF1		
	RS2 + LS2 + RA2 + LA		
	RA2 + LA2 + RF2 + LF2		
	RS1 + LS1 + RF1 + LF1		
Inter-Silhouette Pentagon	H1 + RS1 + LS1 + RA1 + LA1	30	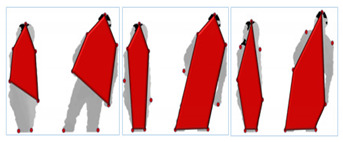
	H1 + RA1 + LA1 + RF1 + LF1		
	H1 + RS1 + LS1 + RF1 + LF1		
	H2 + RS2 + LS2 + RA2 + LA		
	H2 + RA2 + LA2 + RF2 + LF2		
	H2 + RS1 + LS1 + RF1 + LF1		
Intra-Silhouette Triangle	H1 + RS2 + LS2	18	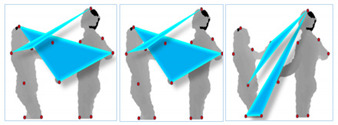
	H1 + RA2 + LA2		
	H1 + RF2 + LF2		
	H2 + RS1 + LS1		
	H2 + RA1 + LA1		
	H2 + RF1 + LF1		
Intra-silhouette Quadrangular	RS1 + LS1 + RS2 + LS2	16	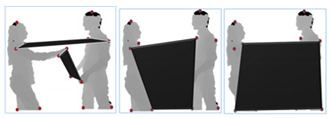
	RA1 + LA1 + RA2 + LA2		
	RS1 + RS2 + RF1 + RF2		
	LS1 + LS2 + LF1 + LF2		
Intra-silhouette Pentagon	H1 + LS1 + RS1 + LF2 + RF2	30	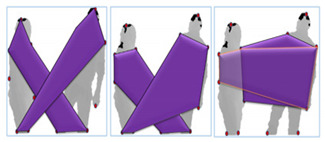
	H2 + LS2 + RS2 + LF1 + RF1		
	LS1 + RS1 + RA1 + LF2 + RF2		
	LS2 + RS2 + LA2 + LF1 + RF1		
	LS1 + RS1 + RA1 + LS2 + LA2		
	LS2 + RS2 + LA2 + LS1 + LA1		
Total 6 types	32 Geometrical Shapes	136 angles	

^1^ Head of first (left) silhouette, ^2^ Right Shoulder of first silhouette, ^3^ Left Shoulder of first silhouette, ^4^ Right Arm of first silhouette, ^5^ Left Arm of first silhouette, ^6^ Right Foot of first silhouette, ^7^ Left Foot of first silhouette, ^8^ Head of second (right) silhouette, ^9^ Right Shoulder of second silhouette, ^10^ Left Shoulder of second silhouette, ^11^ Right Arm of second silhouette, ^12^ Left Arm of second silhouette, ^13^ Right Foot of second silhouette and ^14^ Left Foot of second silhouette.

**Table 2:** Confusion matrix showing accuracies over interaction classes of SBU dataset.

Interaction Classes	Approaching	Departing	Kicking	Punching	Pushing	Hugging	Exchanging Object	Shaking Hands
Approaching	**0.92**	0.04	0	0	0	0	0.02	0.02
Departing	0	**0.95**	0	0	0.03	0.01	0.01	0
Kicking	0	0	**0.98**	0.02	0	0	0	0
Punching	0.02	0	0.03	**0.89**	0.05	0.01	0	0
Pushing	0.01	0.02	0	0.05	**0.88**	0.03	0	0.01
Hugging	0.01	0	0	0.02	0.02	**0.95**	0	0
Exchanging Object	0.04	0	0	0	0	0.02	**0.86**	0.08
Shaking Hands	0.05	0	0	0	0	0.01	0.07	**0.87**
**Mean Recognition Accuracy rate = 91.25%**								

**Table 3:** Confusion matrix showing accuracies over interaction classes of UoL dataset.

Interaction Classes	Handshake	Hug	Help Walk	Help Stand-up	Fight	Push	Conversation	Call Attention
Handshake	**0.85**	0	0.05	0.07	0	0	0.03	0
Hug	0	**0.93**	0.06	0	0	0.01	0	0
Help walk	0.02	0.05	**0.89**	0.04	0	0	0	0
Help stand-up	0.09	0	0	**0.87**	0	0.04	0	0
Fight	0	0	0	0	**0.95**	0.03	0.02	0
Push	0	0	0.01	0	0.07	**0.91**	0.01	0
Conversation	0.02	0	0	0	0	0	**0.91**	0.07
Call Attention	0	0	0	0	0	0	0.08	**0.92**
**Mean Recognition Accuracy rate = 90.4%**								

**Table 4:** Confusion matrix showing accuracies over interaction classes of UT-Interaction Set 1.

Interaction Classes	Shake Hands	Point	Hug	Push	Kick	Punch
Shake Hands	**0.93**	0.05	0	0.02	0	0
Point	0.04	**0.88**	0	0.05	0	0.03
Hug	0.03	0	**0.89**	0.05	0	0.03
Push	0	0.03	0.03	**0.84**	0	0.10
Kick	0	0.02	0	0	**0.90**	0.08
Punch	0.02	0.03	0	0.08	0.03	**0.84**
**Mean Recognition Accuracy rate = 88.0%**						

**Table 5:** Confusion matrix showing accuracies over interaction classes of UT-Interaction Set 2.

Interaction Classes	Shake Hands	Point	Hug	Push	Kick	Punch
Shake Hands	**0.90**	0.05	0	0.03	0	0.02
Point	0.05	**0.87**	0	0.04	0	0.04
Hug	0.02	0	**0.88**	0.06	0	0.04
Push	0	0.01	0.03	**0.85**	0	0.11
Kick	0	0	0.01	0.04	**0.89**	0.06
Punch	0.04	0.05	0	0.09	0	**0.82**
**Mean Recognition Accuracy rate = 86.8%**						

**Table 6:** Comparison of precision, recall and F1 score over three benchmark datasets.

Datasets	Interactions	Precision	Recall	F1 Score
SBU	Approaching	0.88	0.92	0.90
	Departing	0.94	0.95	0.95
	Kicking	0.97	0.98	0.98
	Punching	0.91	0.89	0.90
	Pushing	0.90	0.88	0.89
	Hugging	0.92	0.95	0.94
	Exchanging Object	0.90	0.86	0.88
	Shaking Hands	0.89	0.87	0.88
**Average**		**91.037**	**91.25**	**91.50**
UoL	Handshake	0.87	0.85	0.86
	Hug	0.95	0.93	0.94
	Help walk	0.88	0.89	0.89
	Help Stand-up	0.89	0.87	0.88
	Fight	0.93	0.95	0.94
	Push	0.92	0.91	0.91
	Conversation	0.87	0.91	0.89
	Call Attention	0.93	0.92	0.92
**Average**		**90.50**	**90.37**	**90.38**
UT-Interactions Set 1	Shake Hands	0.91	0.93	0.92
	Point	0.87	0.88	0.88
	Hug	0.97	0.89	0.93
	Push	0.81	0.84	0.82
	Kick	0.97	0.90	0.93
	Punch	0.78	0.84	0.81
**Average**		**88.50**	**88.0**	**88.16**
UT-Interactions Set 2	Shake Hands	0.89	0.9	0.9
	Point	0.89	0.87	0.88
	Hug	0.96	0.88	0.92
	Push	0.77	0.85	0.81
	Kick	1.0	0.89	0.94
	Punch	0.75	0.82	0.78
**Average**		**87.66**	**86.83**	**87.16**

**Table 7:** Comparison of number of states and observations of MEMM over SBU dataset.

Parameters		Performance	
Number of States	Observations	Computational Time (sec)	Accuracy (%)
**4**	X = 10	15.5	76.8
	X = 20	18.3	78.5
	X = 30	23	79.2
**5**	X = 10	21.5	82.8
	X = 20	26.4	83
	X = 30	30.8	86
**6**	X = 10	22.2	88.6
	X = 20	30.6	89
	X = 30	37.9	91.20

**Table 8:** Comparison of number of states and observations of MEMM over UoL dataset.

Parameters		Performance	
Number of states	Observations	Computational time (sec)	Accuracy (%)
**4**	X = 15	17.5	78
	X = 25	25.3	80.5
	X = 35	28.5	81.9
**5**	X = 15	25.2	83.8
	X = 25	32.9	86
	X = 35	40.0	88
**6**	X = 15	45.2	88.9.6
	X = 25	48.2	90
	X = 35	49	90.8

**Table 9:** Comparison of number of states and observations of MEMM over UT-Interaction dataset.

Parameters		Performance	
Number of States	Observations	Computational Time (sec)	Accuracy (%)
**3**	X = 10	10.2	69.8
	X = 20	12.1	70.2
	X = 30	15	70.9
**4**	X = 10	14	74.2
	X = 20	16.4	76.3
	X = 30	18.8	78.0
**5**	X = 10	22.4	82.5
	X = 20	25.7	85.2
	X = 30	38.0	88.5

**Table 10:** Comparison of proposed hybrid features HIR system with other state-of-the-art systems over SBU interaction dataset.

Methods	HIR Accuracy (%)			
	SBU Dataset	UoL Dataset	UT Interaction Set 1	UT Interaction Set 2
CFDM [81]	89.4			
CWDTW [82]	90.8			
CHARM [83]	84			
Joint Features [84]	90.3			
Body parts contrast mining [85]	86.9			
Deep LSTM [86]	90.41			
Skeletal data [87]	88	87		
Skeletal and Geometrical features [88]	-	85.56		
Spatio–temporal+ social features [30]	-	85.12		
Spatio–temporal features [89]			83.5	72.5
SPN Graph [90]			82.4	85.3
Discriminative model [91]			85	85
**Proposed hybrid feature**	**91.25**	**90.4**	**88** **.0**	**86.8**
